# HIV/AIDS patients undergoing total knee arthroplasty are at higher risk of acute renal failure and transfusion and incurred higher cost: a propensity-matched database study

**DOI:** 10.1186/s43019-022-00156-0

**Published:** 2022-06-15

**Authors:** Vishaal Sakthivelnathan, Tejas Senthil, Sushrruti Varatharaj, Varatharaj Mounasamy, Senthil Sambandam

**Affiliations:** 1 School of Medicine, University of Texas Medical Branch, 301 University Boulevard, Galveston, TX 77555 USA; 2 Carroll High School, Southlake, TX USA; 3 Burrell College of Osteopathic Medicine, Las Cruces, NM USA; 4 Department of Orthopedics, University of Texas Southwestern, Dallas VAMC, Dallas, TX USA; 5 University of Texas Southwestern, Dallas VAMC, Dallas, TX USA

**Keywords:** HIV, AIDS, Total knee arthroplasty

## Abstract

**Introduction:**

Human immunodeficiency virus (HIV) is a retrovirus that can cause acquired immunodeficiency syndrome (AIDS). Total knee arthroplasty (TKA) in HIV-positive patients has not been well documented in the current literature. Thus, this study aimed to examine the early postoperative outcomes and complications of HIV-positive TKA patients as compared to TKA patients who are HIV-negative patients by utilizing the National Inpatient Sample (NIS) database.

**Methods:**

Admissions data for TKA and HIV were analyzed from the NIS database using ICD-10-CM diagnosis codes. An extensive array of preoperative and postoperative variables was compared among HIV positive TKA patients and HIV negative TKA patients. An unmatched analysis and a matched analysis using a 1:1 propensity match algorithm were conducted to compare the two groups.

**Results:**

The average age of the HIV-positive group was lower than the HIV-negative group (59.0 vs 66.7, *p* < 0.001). The HIV-positive group had a smaller percentage of females (38.4% vs 61.5%, *p* < 0.001) and a lower incidence of tobacco-related disorders than the HIV-negative group (10.3% vs 15.8%, *p* = 0.032). The HIV-positive group had a longer mean length of stay (3.0 days vs 2.4 days, *p* < 0.001) and a greater mean total charge incurred (90,780.25 vs 64,801.55, *p* < 0.001). In the unmatched analysis, the incidence of acute renal failure (6.4% vs 2%, *p* < 0.001), transfusions (3.9% vs 1.5%, *p* = 0.004), and periprosthetic joint infection (3% vs 1%, *p* = 0.007) was higher in HIV positive group. The matched analysis showed a higher incidence of acute renal failure group (6.4% vs 0.5%, *p* = 0.01) and transfusions (3.9% vs 5%, *p* = 0.01) in the HIV-positive but a statistically insignificant difference in the rate of periprosthetic joint infection (3% vs 1%, *p* = 0.153).

**Conclusion:**

HIV/AIDS is associated with an increased incidence of acute renal failure and transfusions, as well as a longer length of stay and higher incurred costs in TKA patients.

## Introduction

Human Immunodeficiency Virus (HIV) is a retrovirus that infects white blood cells and can cause Acquired Immunodeficiency Syndrome (AIDS), a condition characterized by a weakened immune system and recurrent opportunistic infections. In 2019, the prevalence of HIV in the United States was estimated to be 1,189,700, with an incidence of 36,801 [1]. Patients with HIV/AIDS can take highly active antiretroviral therapy (HAART), which has been shown to improve life expectancy to a level approaching that of the general population [2, 3].

Total knee arthroplasty (TKA) is a procedure indicated in patients with osteoarthritis, rheumatoid arthritis, and degenerative joint disease of the knee joint cartilage. A recent longitudinal study found that the knee cartilage matrices in patients with HIV receiving HAART were more disordered and heterogenous [4]. This finding, along with the increased longevity of HIV patients as a result of HAART, warrants further investigation of TKA outcomes and complications in the HIV population.

There is a lack of studies comparing the rates of complications in TKA patients with HIV/AIDS compared to those without HIV/AIDS, with the limited current literature presenting conflicting evidence [5]. The purpose of this study was to assess the postoperative outcomes following TKA in patients with HIV/AIDS as compared to HIV negative TKA patients using the National Inpatient Sample (NIS) database from the Healthcare Cost and Utilization Project (HCUP) [6]. We hypothesized that TKA patients with HIV are at higher risk of local and systemic complications and likely to incur more cost.

## Methods

### Database description

This study was completed using NIS database data from the years 2016 to 2019. This timeframe was selected since 2016 was the first full year that HCUP transitioned to the International Classification of Diseases 10th Revision classification system. The NIS database is a component of the Healthcare Cost and Utilization Project (HCUP) It is the largest all-payer inpatient care database within the United States, with data from more than 7 million hospital stays per year. The data encompasses 20% of the hospitals in the United States and is verified using a quality assessment evaluation comparing data points to standardized normative values by an independent contractor.

Data elements within the NIS Database include demographic data, length of stay, source of payment, hospital charges, discharge status, comorbidities, and more. The 2016–2019 version utilizes the International Classification of Diseases, Tenth Revision, Clinical Modification/Procedure Coding System (ICD-10-CM/PCS).

### Data acquisition

This study was exempt from IRB approval since the data was de-identified and publicly available. Patients who underwent TKA were identified using the ICD-10 procedural codes OSRC and OSRD (Table 1). Data from patients with HIV/AIDS were extracted using the ICD codes B20, B21, B22, B23, B24 (Table 1).

**Table 1:** ICD 10 codes used

Total knee arthroplasty procedure code	HIV	Obese codes	Comorbidities codes	Medical complications codes	Surgical complications codes
Replacement of Left Knee Joint OSRD Replacement of Right Knee Joint OSRC	B20 B21 B22 B23 B24 B25	E660 E6601 E6609 E661 E662 E668 E669 Z6830 Z6831 Z6832 Z6833 Z6834 Z6835 Z6836 Z6837 Z6838 Z6839	Diabetes without complications E119 Diabetes with complications E1169 Tobacco related disorder Z87891	Acute renal Failure N170, N171, N172, N178, N179 Myocardial Infarction I2101, I2102, I2111, I2113, I12114, I12119, I2121, I12129, I21A1 Blood loss anemia D62 Pneumonia J189, J159, J22 Blood transfusion 30233N1 Pulmonary embolism I2602, I2609, I2692, I2699 Deep Vein Thrombosis I82401,I82402, I82403, I82409, I82411, I82412, I82413, I82419, I82421, I82422, I82423, I82429, I82431, I82432, I82433, I82439, I82441, I82442, I82443, I82449, I82491, I82492, I82493, I82499, I824Y1, I824Y2, I824Y3, I824Y9, I824Z1, I824Z2, I824Z3, I824Z4	Periprosthetic fracture T84010A, T84011A, T84012A, T84013A, T84018A, T84019A, M9665, M96661, M96662, M96669, M96671, M96672, M96679, M9669, M9701XA, M9702XA, M9711XA, M9712XA Periprosthetic dislocation T84020A, T84021A, T84022A, T84023A, T84028A, T84029A Periprosthetic mechanical complications Periprosthetic fracture T84090A, T84091A, T84092A, T84093A, T84098A, T84099A Periprosthetic Infection T8450XA, T8451XA, T8452XA, T8453XA, T8454XA, T8459XA Superficial Surgical Site Infection T8141XA Deep Surgical Site Infection T8142XA Wound Dehiscence T8130XA, T8131XA, T8132XA

Pre-operative variables included (1) age (2) sex (3) elective vs non-elective (4) diabetes without complications (5) tobacco use disorder, and (6) obesity (Table 1). Post-operative medical and surgical outcomes aggregated from the NIS database include (1) length of stay (2) total incurred charges (3) mortality (4) elective vs non-elective admission (5) acute renal failure (6) myocardial infarction (7) blood loss anemia (8) pneumonia 9) pulmonary embolism (10) deep vein thrombosis (11) periprosthetic fracture (12) periprosthetic dislocation (13) periprosthetic mechanical complication (14) periprosthetic infection (15) superficial surgical site infection (16) deep surgical site infection (17) wound dehiscence (18) cardiac arrest and ventricular fibrillation and (19) blood transfusion (Table 1). These complications are representative of the most common complications of TKA available on the NIS database.

### Statistical analysis

All statistical analyses were conducted using SPSS version 27.0 (IBM; Armonk, NY, USA). Originally, descriptive statistics were used to aggregate patient demographic data. An unmatched analysis and matched analysis were completed. A 1:1 propensity matching algorithm using the pre-operative was performed. *T*-tests were used when analyzing numerical variables. Chi-squared analyses were used when analyzing binomial variables. Fischer Exact tests were used when the incidence values were less than 5. A *p*-value < 0.05 was considered statistically significant for all tests. Odds ratios and their corresponding 95% confidence intervals for the surgical outcomes and complications were measured as a ratio of the incidence in the HIV/AIDS group to the incidence in the HIV-negative control group.

## Results

**Fig. 1:**
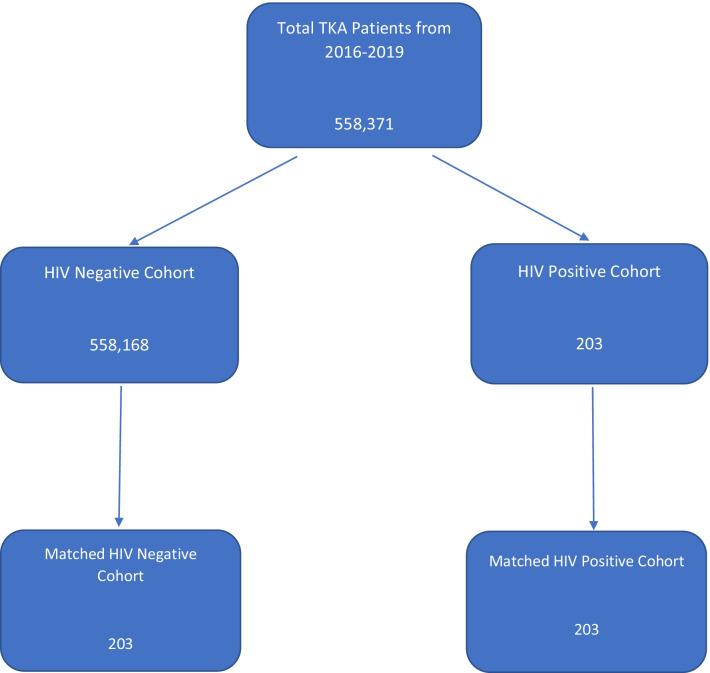
Patient data selection algorithm

A total of 558,371 patients who underwent TKA were identified using the NIS database. Within that population, 558,168 patients did not have HIV/AIDS and 203 patients had HIV/AIDS (Fig. 1).

### Demographic data

On average, the patients in the HIV/AIDS groups, with a mean age of 58.9 years, were younger than the control group, with a mean age of 66.7 years (*p* < 0.001). The HIV group tended to have fewer females, with the percentage of females being 38.4%, than the control group, with the percentage of females being 61.5% (*p* < 0.001). The incidence of tobacco-related disorders was lower in the HIV group (10.3%) than in the control group (15.8%, *p* = 0.032). The other demographic variables were not significantly different among the two groups (Table 2).

**Table 2:** Patient Demographics

	HIV group	Control group	Odds Ratio (HIV group/Control group)	Odds ratio 95% confidence interval	Significance
Pre operative variables					
Mean age (standard deviation) in years*	58.99 (8.699)	66.72 (9.504)	–	–	*p* < 0.001
Sex (percentage female)*	38.4%	61.5%	0.390	[0.294, 0.518]	*p* < 0.001
Elective vs Non-elective admission (percentage elective)	95.5%	96%	0.883	[0.452, 1.722]	*p* = 0.714
Diabetes without complication (percentage diabetic)	12.3%	14.8%	0.810	[0.533, 1.232]	*p* = 0.324
Tobacco use disorder (percentage users)*	10.3%	15.8%	0.614	[0.391, 0.964]	*p* = 0.032
Obesity (percentage obese)	34%	30.9%	1.150	[0.860, 1.538]	*p* = 0.344

### Unmatched post operative outcomes analysis

The incidence of acute renal failure was greater in the HIV group, 6.4%, than in the control group, 2%, (*p* < 0.001). The incidence of periprosthetic fractures was greater in the HIV group, 3%, than in the control group, 1% (*p* = 0.007). The incidence of blood transfusions was greater in the HIV group, 3.9%, than in the control group, 1.5% (*p* = 0.004). The other postoperative outcomes showed no significant differences among the two groups in the unmatched analysis (Table 3).

**Table 3:** Unmatched analysis

	HIV group	Control group	Odds Ratio (HIV group/Control group)	Odds ratio 95% confidence interval	Significance
Post operative variables (Incidence percentage)					
Mortality	0	0. 04%	1	[1, 1]	*p* = 0.789
Acute renal failure*	6.4%	2%	3.381	[1.927, 5.932]	*p* < 0.001
Myocardial infarction	0%	0.02%	1	[1, 1]	*p* = 0.842
Blood loss anemia	16.7%	15.3%	1.112	[0.769, 1.607]	*p* = 0.573
Pneumonia	0.5%	0.2%	2.537	[0.355, 18.115]	*p* = 0.336
Pulmonary embolism	0.5%	0.2%	2.229	[0.312, 15.912]	*p* = 0.412
Deep vein thrombosis	0.5%	0.2%	2.186	[0.306, 15.608]	*p* = 0.424
Periprosthetic fracture	1%	0.4%	2.342	[0.581, 9.436]	*p* = 0.217
Periprosthetic dislocation	1%	1%	1.294	[0.321, 5.211]	*p* = 0.716
Periprosthetic mechanical complication	1.5%	0.8%	1.845	[0.590, 5.772]	*p* = 0.285
Periprosthetic infection*	3%	1%	2.903	[1.288, 6.542]	*p* = 0.007
Superficial surgical site infection	0%	0%	1	[1, 1]	*p* = 0.923
Deep surgical site infection	0%	0%	1	[1, 1]	*p* = 0.957
Wound dehiscence	0.5%	0.1%	5.219	[0.730, 37.295]	*p* = 0.066
Cardiac arrest and ventricular fibrillation	0%	0%	1	[1, 1]	*p* = 0.923
Blood transfusion*	3.9%	1.5%	2.736	[1.349, 5.550]	*p* = 0.004

*Statistically significant

The average total incurred charge for the HIV group was higher in the HIV group, with a mean of $90,740.25 and a standard deviation of $88,045.91, than in the control group, with a mean of 64,801.55 and a standard deviation of $45,809.89 (*p* < 0.001). The average length of stay was longer in the HIV group, with a mean of 3.0 days and a standard deviation of 3.2 days, than in the control group, with a mean of 2.4 days and a standard deviation of 1.9 days (*p* < 0.001).

### Matched post operative outcomes analysis

The 1:1 propensity match algorithm yielded 203 patients in the HIV group and 203 patients in the control group. The incidence of acute renal failure was greater in the HIV group, 6.4%, than in the control group, 0.5% (*p* ≤ 0.01). The incidence of blood transfusions was greater in the HIV group, 3.9%, than in the control group, 0.5% (*p* = 0.018). The other post-operative outcomes showed no significant differences among the two groups in the matched analysis (Table 4).

**Table 4:** Matched Analysis

	HIV group	Control group	Odds Ratio (HIV group / Control group)	Odds Ratio 95% Confidence Interval	Significance
Post operative variables (Incidence percentage)					
Mortality	0	0	**	**	**
Acute renal failure*	6.4%	0.5%	13.821	[1.791, 106.673]	*p* = 0.01
Myocardial infarction	0%	0%	**	**	**
Blood loss anemia	16.7%	9.9%	1.841	[1.020, 3.323]	*p* = 0.041
Pneumonia	0.5%	0	0.499	[0.452, 0.550]	*p* = 0.499
Pulmonary embolism	0.5%	1%	0.498	[0.045, 5.530]	*p* = 0.562
Deep vein thrombosis	0.5%	0.5%	1	[0.062, 16.097]	*p* = 1.000
Periprosthetic fracture	1%	1%	1	[0.139, 7.168]	*p* = 1.000
Periprosthetic dislocation	1%	0.5%	2.010	[0.181, 22.343]	*p* = 0.562
Periprosthetic mechanical complication	1.5%	0%	0.496	[0.450, 0.548]	*p* = 0.082
Periprosthetic infection	3%	1%	3.061	[0.610, 15.349]	*p* = 0.153
Superficial surgical site infection	0%	0%	**	**	**
Deep surgical site infection	0%	0%	**	**	**
Wound dehiscence	0.5%	0.5%	1	[0.062, 16.097]	*p* = 1
Cardiac arrest and ventricular fibrillation	0%	0%	**	**	**
Blood transfusion*	3.9%	0.5%	8.287	[1.027, 66.827]	*p* = 0.018

*Statistically significant

**Because incidence was 0 in one or both groups, odds ratio and significance values could not be calculated

The average total incurred charges for the HIV group was higher in the HIV group, with a mean of $90,740.25 and a standard deviation of $88,045.91, than in the control group, with a mean of 43,233.93 and a standard deviation of $14,625.30 (*p* < 0.001). The average length of stay was longer in the HIV group, with a mean of 3.0 days and a standard deviation of 3.2 days, than in the control group, with a mean of 2.8 days and a standard deviation of 2.0 days (*p* < 0.001).

## Discussion

Data from the matched outcomes analysis indicated that patients with HIV/AIDS who had TKA had a significantly higher incidence of blood transfusions and acute renal failure than TKA patients who are HIV negative. Additionally, the mean total incurred charges and the length of stay were greater in the HIV/AIDS group. There were no other significant differences among the other postoperative variables, including mortality.

The relationship between TKA in HIV/AIDS patients and length of stay has been reported by other studies. Boylan et al. reported that the average length of stay for HIV/AIDS patients who had TKA was 17% longer than that of TKA patients without HIV/AIDS [7]. Roof et al. conducted a multicenter retrospective case–control study that reported an average length of stay of 3.8 days for TKA patients with HIV/AIDS as compared to 2.28 days in TKA patients without HIV/AIDS [8]. Although there are no current studies that have published total charges incurred, the increased average length of stay in HIV/AIDS patients is likely a contributing factor.

In our study, the incidence of acute renal failure was higher in the HIV/AIDS group compared to the group without HIV/AIDS. In a retrospective cohort study of, Akkaya et al. reported 1 patient out of 11 HIV positive TKA patients developing acute renal failure [9]. A study by Lin et al. has reported the rate of kidney failure in joint replacement patients with HIV/AIDS a higher incidence of acute renal failure in HIV/AIDS patients (4.2%) who underwent joint replacement than in HIV-negative patients (1.6%) [10]. The study mentioned that abnormal weight loss and malnutrition, fluid and electrolyte imbalances, and chronic kidney disease had a stronger association with post-operative complications, as a broad category, than HIV status, but did not report if this specifically included acute renal failure. One explanation for the increase in acute kidney injury seen in the HIV cohort could include the nephrotoxicity of many HAART agents [11]. Pre-operative renal damage as a result of HAART could predispose the HIV cohort to further any renal insults due to surgical procedures.

The unmatched analysis found a significantly greater incidence of periprosthetic joint infection in the HIV/AIDS group, but this was not supported by the matched analysis. A meta-analysis by O’Neil et al. found a total risk ratio of 3.31 for postoperative infection in HIV-positive patients who underwent TKA as compared to HIV negative patients [5]. Lin et al. reported an increased risk of postoperative wound infection in HIV/AIDS patients who had a joint replacement, but also noted that other variables such as abnormal weight loss and malnutrition, fluid and electrolyte imbalances, and chronic kidney disease had a stronger association with complications than HIV status [10]. A retrospective cohort study by Falakassa et al. reported a similar risk for periprosthetic joint infection in HIV patients on highly active antiretroviral therapy (HAART) as compared to HIV negative patients [12]. Capogna et al. conducted a retrospective cohort study that found a nonsignificant difference in the infection rate among HIV/AIDS patients and HIV negative patients who underwent joint replacement (4.4% as compared to 0.7%), claiming that this increased incidence could be attributed to IV drug usage among their HIV cohort [13]. A systematic review by Enayatollahi et al. suggests that highly active antiretroviral therapy is associated with a decreased infection rate in joint replacement patients, with an odds ratio of 0.12 [14]. Our data in conjunction with the current literature seems to indicate that the risk of periprosthetic infection is not increased in HIV patients when controlled for other complications and comorbidities.

This study also found that HIV/AIDS patients who had TKA were more likely to have needed a transfusion post-surgery than HIV negative patients. Although no other studies currently have corroborated and explained this finding as it specifically relates to TKA, in general, cytopenia can be more commonly observed in HIV patients [15]. The incidence of anemia increases as the disease progresses [16]. Since the ICD codes used to extract patient data selected for patients who had AIDS-defining illnesses which are more common later in the disease progression, it is reasonable to assume that these patients were at higher risk of anemias, leading to an increased incidence of post-operative transfusions.

This study is not without limitations. First, the ICD 10 classification for HIV/AIDS categorizes the condition based on the presence of AIDS-defining illnesses, instead of the stage of HIV. Although AIDS-defining illnesses occur later in disease progression, this study could be improved if we were able to categorize patients by HIV staging or CD4 count. Also, this study only considers inpatient data and not longitudinal data. Thus, we were unable to determine if there were any significant differences in 1 month, 3 month, 1 year, and long-term mortality rates or analyze other long-term complications. Additionally, the NIS database only represents 20% of the community hospitals in the United States. Nevertheless, the high volume of data strengthens its generalizability to the U.S. population. Another limitation is a result of the data collection and input method, as the data is subject to coding or reporting errors. Finally, the study period was only 3 years as a result of the transition to a newer classification system in 2016. Although this provided with more stratified data, the sample size was limited.

## Conclusion

Our study found that early postoperative acute renal failure and transfusions were more common in HIV/AIDS patients who had TKA as compared to HIV-negative TKA patients. Additionally, the average length of stay and average total incurred charges were higher in the HIV-positive group. Thus, it is important for surgeons to consider HIV status as well as other comorbidities when performing TKA, especially in hospitals with limited support services and resources.

## Data Availability

Publicly available data through the national inpatient sample database from the healthcare cost and utilization project.
